# Navigating challenges in pediatric trial conduct: integrating bayesian sequential design with semiparametric elicitation for handling primary and secondary endpoints

**DOI:** 10.1186/s12874-025-02484-7

**Published:** 2025-03-31

**Authors:** Danila Azzolina, Ileana Baldi, Silvia Bressan, Mohd Rashid Khan, Liviana Da Dalt, Dario Gregori, Paola Berchialla

**Affiliations:** 1 Department of Environmental and Preventive Science, University of Ferrara, Ferrara, Italy; 2 Clinical Trial and Biostatistics, Research and Development Unit, University Hospital of Ferrara, Ferrara, Italy; 3 Unit of Biostatistics, Epidemiology and Public Health, Department of Cardiac, Thoracic, Vascular Sciences and Public Health, University of Padova, Via Loredan 18, Padova, 35131 Italy; 4 Department of Women’s and Children’s Health, University of Padova, Padova, Italy; 5 Department of Clinical and Biological Science, University of Turin, Turin, Italy

**Keywords:** Pediatric trial, Semiparametric prior, Prior Elicitation, Multiple endpoints, Sequential design, Bayesian trial

## Abstract

**Background:**

This study presents a Bayesian Adaptive Semiparametric approach designed to address the challenges of pediatric randomized controlled trials (RCTs). The study focuses on efficiently handling primary and secondary endpoints, a critical aspect often overlooked in pediatric trials. This methodology is particularly pertinent in scenarios where sparse or conflicting prior data are present, a common occurrence in pediatric research, particularly for rare diseases or conditions.

**Method:**

Our approach considers Bayesian adaptive design, enhanced with B-Spline Semiparametric priors, allowing for the dynamic updating of priors with ongoing data. This improves the efficiency and accuracy of the treatment effect estimation. The Semiparametric prior inherent flexibility makes it suitable for pediatric populations, where responses to treatment can be highly variable. The design operative characteristics were assessed through a simulation study, motivated by the real-world case of the REnal SCarring Urinary infEction Trial (RESCUE).

**Result:**

We demonstrate that Semiparametric prior parametrization exhibits an improved tendency to correctly declare the treatment effect at the study conclusion, even if recruitment challenges, uncertainty, and prior-data conflict arise. Moreover, the Semiparametric prior design demonstrates an improved ability in truly stopping for futility, with this tendency varying with the sample size and discontinuation rates. Approaches based on Parametric priors are more effective in detecting treatment efficacy during interim assessments, particularly with larger sample sizes.

**Conclusion:**

Our findings indicate that these methods are especially effective in managing the complexities of pediatric trials, where prior data may be limited or contradictory. The flexibility of Semiparametric prior design in incorporating new evidence proves advantageous in addressing recruitment challenges and making informed decisions with restricted data.

**Supplementary Information:**

The online version contains supplementary material available at 10.1186/s12874-025-02484-7.

## Introduction

Pediatric clinical trials are fraught with unique challenges that set them apart from adult trials [1]. Among these challenges is the ethical dilemma posed by the involvement of vulnerable populations. The requirement for minimal risk and maximal benefit is stringent, considering the developing physiology and psychology of pediatric patients [2] as recognized by the U.S. Food and Drug Administration (FDA) [3]. Moreover, the small populations for many pediatric conditions necessitate extremely efficient and ethically sound trial designs. The FDA and European Medicines Agency (EMA) have recognized the difficulty in recruiting pediatric participants, often due to the rarity of some pediatric diseases and the reluctance of parents to enroll their children in clinical trials [4, 5]. To address these challenges, agencies advocate innovative trial designs that maximize data yield while minimizing the number of participants and exposure to potential risks [6]. In this regard, adaptive trial designs, which offer the flexibility to modify key design aspects based on interim data, align with the ethical mandate to reduce patient burden and improve trial efficiency [7].

Traditional clinical trial methodologies often fall short in the pediatric context owing to their rigidity and assumptions, which may not hold for children [8]. For instance, the linear dose-response relationship assumed in many adult trials may not apply to pediatric populations, where developmental stages can significantly affect treatment efficacy and safety [9]. Moreover, the need to minimize patient burden and ethical concerns about placebo use makes it essential to derive conclusive results from smaller sample sizes and shorter trial durations [10].

Another challenge in pediatric trials is the incorporation of secondary endpoints, particularly discontinuation rates. These rates provide useful information for treatment tolerability and long-term feasibility, which are important in pediatric settings where long-term compliance is a major concern [11]. However, the limited sample sizes and the challenges in study conduction make it less feasible to address these endpoints using traditional techniques for multiple testing to control the false discovery rate [12]. In several cases, combining several secondary endpoints into a single composite outcome is an alternative that can increase the efficiency of statistical analyses, especially in trials with small sample sizes [12]. When a composite endpoint yields a significant result, it is not automatically applied to each component. Therefore, interpreting the outcome of a test based on a composite endpoint is straightforward only when we assume that all the components are uniformly affected [13]. In this general framework, the literature suggests using advanced adaptive and/or Bayesian [14] design to address secondary endpoints, allowing for modifications to the trial based on interim analyses, especially when dealing with a limited sample size [15]. This flexibility can help to address unexpected issues related to tolerability or compliance.

Accounting for these challenges, using a Bayesian Sequential design with a secondary endpoint interim assessment and a Semiparametric prior elicitation could be a proficient strategy for trial design. This methodological approach is useful when the data for defining priors are uncertain or a prior-data conflict can arise [16]. Other approaches are also suggested in the literature for handling uncertainty and prior data conflicts. For example, the Hierarchical Bayesian models, or nonparametric priors [17] can be a robust framework for incorporating data from multiple levels of hierarchy and handling variability across different patient groups [18]. However, some model parametrizations often demand detailed information for reliable treatment effect estimations across different levels, which is challenging in pediatric trials due to the challenges in enrollment and trial conduction [19]. Additionally, the inherent complexity of these models can result in computationally demanding analyses [20]. The decision to focus on Semiparametric priors in our study was driven by specific considerations highlighted in the literature [16, 21–23]. This approach is particularly relevant when dealing with high uncertainty about pre-trial beliefs on expected response rates, as Semiparametric prior balance flexibility and computational tractability. Moreover, Semiparametric priors can be particularly advantageous in settings where the prior information is uncertain or highly variable, as they allow for constructing priors that more naturally accommodate the range of expert opinions and historical data. The inherent flexibility of Semiparametric prior design to model data without the limitations of strict Parametric rules makes it useful for pediatric populations, where responses to treatments can be highly variable like in a pediatric setting [8, 24].

Moreover, we introduced a Bayesian stopping rule for this design based on the Highest Posterior Density Interval (HDI) coverage for the treatment effect. This method is also used in contexts such as vaccine safety surveillance [25]. Classical stopping boundaries, such as those defined by O’Brien and Fleming [26], optimize Type I and Type II error rates through repeated experiments in a frequentist setting. The Bayesian approach reduces uncertainty regarding the treatment effect by incorporating available data, literature, or expert opinions into the prior distribution. HDI-based decision-making is particularly useful in pediatric trials with limited data and strict ethical considerations, as it continuously updates the probability distribution of the treatment effect, ensuring clear and informed decisions [27].

This article proposes A Bayesian sequential design, as suggested by Gajewski et al. in 2022 [28], enhanced with B-Spline Semiparametric priors [21] and flexible integration of historical data or expert opinions [29] on treatment effects [16] together with a secondary safety endpoint interim assessment. Moreover, the approach integrates the method with the HDI Bayesian-based stopping rules.

We assessed our method’s properties through a simulation study, applying it to various scenarios in a two-endpoint adaptive trial design. This design prioritizes the primary endpoint while ensuring substantial data for the secondary analysis. The simulation study is inspired by a real motivating example: the REnal SCarring Urinary infEction Trial (RESCUE), a pediatric RCT [30] with multiple conduction challenges.

## Methods

### Bayesian two-endpoint adaptive sequential design

**Fig. 1:**
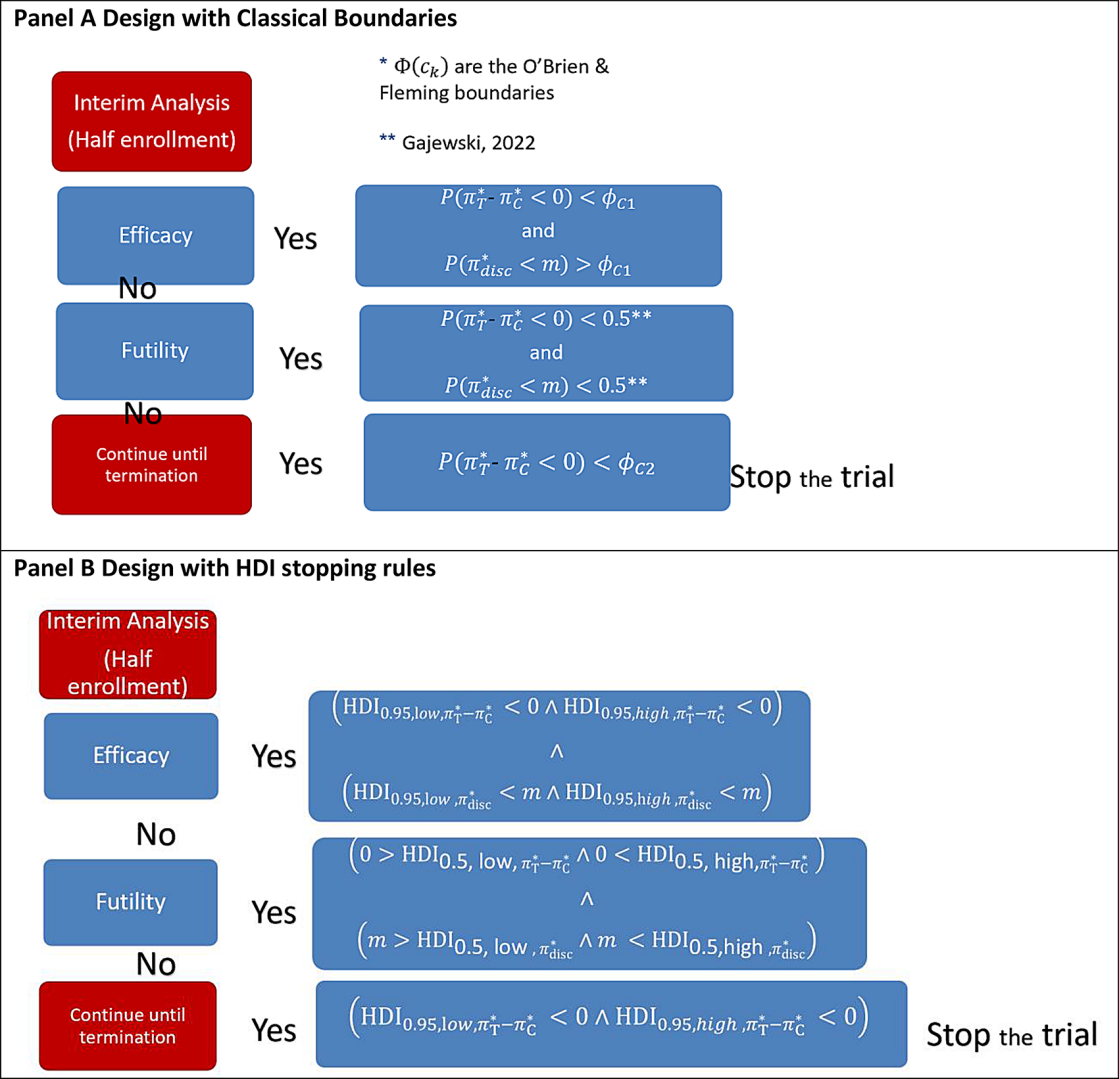
Trial design flowchart

The Bayesian two-endpoint sequential design, as proposed by Gajewski et al. [28], is based on an interim assessment of the treatment effect estimation, together with a secondary endpoint evaluation. A flowchart of the trial design is shown in Fig. 1. At mid-enrolment, an interim evaluation is conducted. The trial stops early for efficacy if the posterior probabilities that the Absolute Risk Reduction (ARR) is below zero and the discontinuation rate (secondary endpoint) is below an acceptability rate *m*, both exceed the stopping boundary.

### Stopping boundary definition

#### Priors

Stopping rules are defined based on posterior probabilities computed using Beta distributions derived from expert elicitation (Appendix A). Semiparametric priors using B-splines were also considered to balance informativeness and robustness in prior data conflicts (Appendix A).

The original design was determined by defining the priors in a Parametric framework [28] considering the normal prior distributions for the treatment effect estimation and secondary endpoint assessment. This study instead used flexible Semiparametric priors for expert opinion elicitation during the study design phase. To investigate the efficacy of the design, a simulation experiment with six prior scenarios (Uninformative, Low-Informative, and Informative in both Parametric and Semiparametric settings) was conducted. The details of the prior distributions are available in the literature [16, 21–23] and Appendix A.

**Classical Stopping Boundaries.** The stopping boundaries were defined by converting the classical frequentist decision thresholds into the cumulative probability of a normal distribution, as reported elsewhere [28]. The posterior ARR is the difference in the event rate between the treatment $\:{\pi\:}_{\text{T}}^{\text{*}}$ and control groups $\:{\pi\:}_{\text{C}}^{\text{*}}$ defined as $P\left(\pi_{\mathrm{T}}^*-\pi_{\mathrm{C}}^*<0\right)>\Phi\left(c_1\right)$. The threshold$\:\varPhi\:\left({c}_{1}\right)=0.997$ is the cumulative probability for a standard random variable defined on the $\:{c}_{1}=\:2.74$ O’Brien-Fleming-like conservative bound. The posterior on the secondary endpoint discontinuation rate is $\:{\pi\:}_{\text{d}\text{i}\text{s}\text{c}}^{\text{*}}$. The trial was also stopped for efficacy if$P\left(\pi_{\text {disc }}^*<\mathrm{m}\right)>\Phi\left(c_1\right)$. For efficacy stopping, both conditions and must be met to ensure robust decision-making [28].                      

At the first interim assessment, the trial can be stopped for futility if $P\left(\pi_{\mathrm{T}}^*-\pi_{\mathrm{C}}^*<0\right)<0.5$ and $\:{P(\pi\:}_{disc}^{\text{*}}<\:\text{m})\:<0.5$. For futility stopping, both conditions must be met simultaneously to ensure a comprehensive evaluation of benefits and safety [28]. The threshold of 0.5, was used by the authors [28] to calibrate the design to have a futility rate of ~ 25% under the scenario of no effect [28].

If the early stopping conditions do not arise, the trial continues until termination, and finally, the efficacy is declared only considering the primary endpoint if$P\left(\pi_{\mathrm{T}}^*-\pi_{\mathrm{C}}^*<0\right)>\Phi\left(c_2\right)$. The final stopping boundary $\:\varPhi\:\left({c}_{2}\right)=\:0.976$ is the cumulative probability of the second interim $\:{c}_{2}=\:2.75$ O’Brien-Fleming-like bound (Fig. 1, Panel A).

**Bayesian HDI Stopping Rules.** The stopping boundaries were also defined by considering the HDI rules (Fig. 1, Panel B). Early rejection for efficacy arises if both the HDI intervals for the difference in rate $\:{\pi\:}_{\text{T}}^{\text{*}}-{\pi\:}_{\text{C}}^{\text{*}}$ exclude 0 and the HDI interval for the discontinuation rate $\:{\pi\:}_{\text{d}\text{i}\text{s}\text{c}}^{\text{*}}$ is lower than the safety threshold $\:\text{m}$:

$$\eqalign{& \left( {{\rm{HD}}{{\rm{I}}_{1 - \varepsilon,\>{\rm{low}}\>,\>\pi \>_{\rm{T}}^{\rm{*}}{\rm{ - }}\pi \>_{\rm{C}}^{\rm{*}}\>}} < 0 \wedge {\rm{HD}}{{\rm{I}}_{1 - \varepsilon,\>{\rm{high}}\>,\>\pi \>_{\rm{T}}^{\rm{*}}{\rm{ - }}\pi \>_{\rm{C}}^{\rm{*}}\>}} < 0} \right) \wedge \cr & \left( {{\rm{HD}}{{\rm{I}}_{1 - \varepsilon,\>\>{\rm{low}}\>,\pi \>_{{\rm{disc}}}^{\rm{*}}}} < m \wedge {\rm{HD}}{{\rm{I}}_{1 - \varepsilon,\>\>{\rm{high}}\>,\pi \>_{{\rm{disc}}}^{\rm{*}}}} < m} \right) \cr} $$

The $\mathrm{HDI}_{1-\varepsilon \text {, low }, \pi_{\mathrm{T}}^*-\pi_{\mathrm{C}}^*}^*$and $\mathrm{HDI}_{1-\varepsilon, \text { high },} \pi_{\mathrm{T}}^*-\pi_{\mathrm{C}}^*$are respectively the lower ($\:\text{low}$) and upper ($\:\text{high}$) bounds of the HDI interval for the posterior ARR $\:{\pi\:}_{\text{T}}^{\text{*}}-{\pi\:}_{\text{C}}^{\text{*}}.\:$ The values $\mathrm{HDI}_{1-\varepsilon, \text { low }}, \pi_{\text {disc }}^*$and $\mathrm{HDI}_{1-\varepsilon, ~ h i g h ~}, \pi_{\text {disc }}^*$are, instead, the lower ($\:\text{low}$) and upper ($\:\text{high}$) extreme of the HDI interval for the posterior discontinuation rate $\:{\pi\:}_{\text{d}\text{i}\text{s}\text{c}}^{\text{*}}.\:$

The HDI coverage was set at $\:0.994$ to mimick the O’Brien-Fleming stopping boundary because$\:\:{c}_{1}=\:2.74$ and $\:\varPhi\:\left({c}_{1}\right)=0.997$ with $\:1-\epsilon\:=1-\left[\left(1-\varPhi\:\left({c}_{1}\right)\right)*2\right]=0.994$.

In this scenario, the interim rejection arises if the posterior HDI for $\:{\pi\:}_{\text{C}}^{\text{*}}-{\pi\:}_{\text{T}}^{\text{*}}$ include the 0 and the posterior HDI interval for the $\:{\pi\:}_{\text{d}\text{i}\text{s}\text{c}}^{\text{*}}$ includes the safety threshold $\:\text{m}$:

$$\eqalign{& \left( {0 > {\rm{HD}}{{\rm{I}}_{{\rm{0}}.{\rm{5}},\>{\rm{low,}}\>\pi \>_{\rm{T}}^{\rm{*}}{\rm{ - }}\pi \>_{\rm{C}}^{\rm{*}}}} \wedge 0 < {\rm{HD}}{{\rm{I}}_{{\rm{0}}.{\rm{5}},\>{\rm{high,}}\pi \>_{\rm{T}}^{\rm{*}}{\rm{ - }}\pi \>_{\rm{C}}^{\rm{*}}\>}}} \right) \wedge \cr & \left( {m > {\rm{HD}}{{\rm{I}}_{{\rm{0}}.{\rm{5}},\>{\rm{low}}\>,\>\pi \>_{{\rm{disc}}}^{\rm{*}}}} \wedge m < {\rm{HD}}{{\rm{I}}_{{\rm{0}}{\rm{.5,high}}\>,\>\>\pi \>_{{\rm{disc}}}^{\rm{*}}}}} \right) \cr} $$

$$\begin{aligned}&\left(0>\operatorname{HDI}_{0.5 \text {, low, }, \pi_{\mathrm{T}}^*-\pi_{\mathrm{C}}^*} \wedge\, 0 < \mathrm{HDI}_{0.5, \text { high }, \pi_{\mathrm{T}}^*-\pi_{\mathrm{C}}^*}\right) \wedge \\ &\left(m>\mathrm{HDI}_{0.5, \text { low }, \pi_{\text {disc }}^*} \wedge m<\mathrm{HDI}_{0.5, \text { high }, \pi_{\text {disc }}^*}\right)\end{aligned}$$

The coverage was set at $\:1-{\upepsilon\:}=0.5$ to mimic the classical stopping criteria.

If interim stopping conditions do not arise, the trial continues until termination. At the end of the trial, efficacy was assessed only on the primary endpoint, stopping if the posterior HDI excluded the 0:

$$\left( {{\rm{HD}}{{\rm{I}}_{{\rm{0}}.{\rm{95}},{\rm{low}}\>,\>\pi \>_{\rm{T}}^{\rm{*}}{\rm{ - }}\pi \>_{\rm{C}}^{\rm{*}}\>}} < 0 \wedge {\rm{HD}}{{\rm{I}}_{0.95,{\rm{high}},\pi \>_{\rm{T}}^{\rm{*}}{\rm{ - }}\pi \>_{\rm{C}}^{\rm{*}}}} < 0} \right)$$

$$\left(\mathrm{HDI}_{0.95, \text { low }, \pi_{\mathrm{T}}^*-\pi_{\mathrm{C}}^*}<0 \wedge \mathrm{HDI}_{0.95, \text { high }, \pi_{\mathrm{T}}^*-\pi_{\mathrm{C}}^*}<0\right)$$

### Motivating example

The design of a phase II clinical trial aimed to evaluate the effect of oral dexamethasone in reducing kidney scars in infants with a first febrile urinary tract infection (UTI) [30] served as a motivating example.

This study evaluated the impact of adding oral steroids to antibiotics in preventing kidney scarring in infants with febrile UTIs compared to antibiotics alone. The primary focus was on scarring rates and the secondary outcome was the acceptability of steroid treatment, assessed by treatment discontinuation and side effects. The protocol identified certain challenges in measuring the outcome (detecting a renal scar via renal scintigraphy) at the 6-month follow-up. Nonetheless, throughout the study period, many participants did not complete the follow-up. This was largely because parents deemed 6-month scintigraphy unnecessary after the acute UTI episode had resolved. Other details are reported in the original publication [30].

### Prior elicitation in RESCUE trial

The prior definitions for this simulation experiment were specifically elicited by following a real expert elicitation experiment conducted within the framework of the RESCUE trial. This methodological choice embeds the simulation in realistic clinical scenarios. The details of the elicitation experiment are reported in Appendix A.

### Simulation plan

A 10,000-run Monte Carlo simulation experiment was conducted to investigate design properties. Several simulation scenarios were provided by mimicking the motivating example data-generation mechanism.

The simulations are based on the outcomes of a real-world clinical trial, the RESCUE trial, as well as a real expert elicitation experiment specifically designed to capture realistic expectations of treatment effects in this context. This elicitation process involved extensive consultations with clinical experts who routinely manage the condition under study, ensuring that our simulation parameters are not only scientifically sound but also clinically relevant.

Simulation plan details are reported in Appendix A.

The design performance has been assessed by considering the following indicators.

1. Proportions of simulated trials declaring the treatment effect at the interim or at the end of the study.
2. Average False Discovery Rate (FDR) over the sample size per simulation scenario.
3. Proportions of simulated trials truly early declaring futility.
4. Proportions of simulated trials falsely declaring futility at the interim assessment.
5. The proportions of simulated trials truly declared efficacy at the interim assessment.

Simulations were performed in R.3.4.2 [31] using RBugs [32] and the SEL [33] package for the Semiparametric elicitation.

## Results

**Fig. 2:**
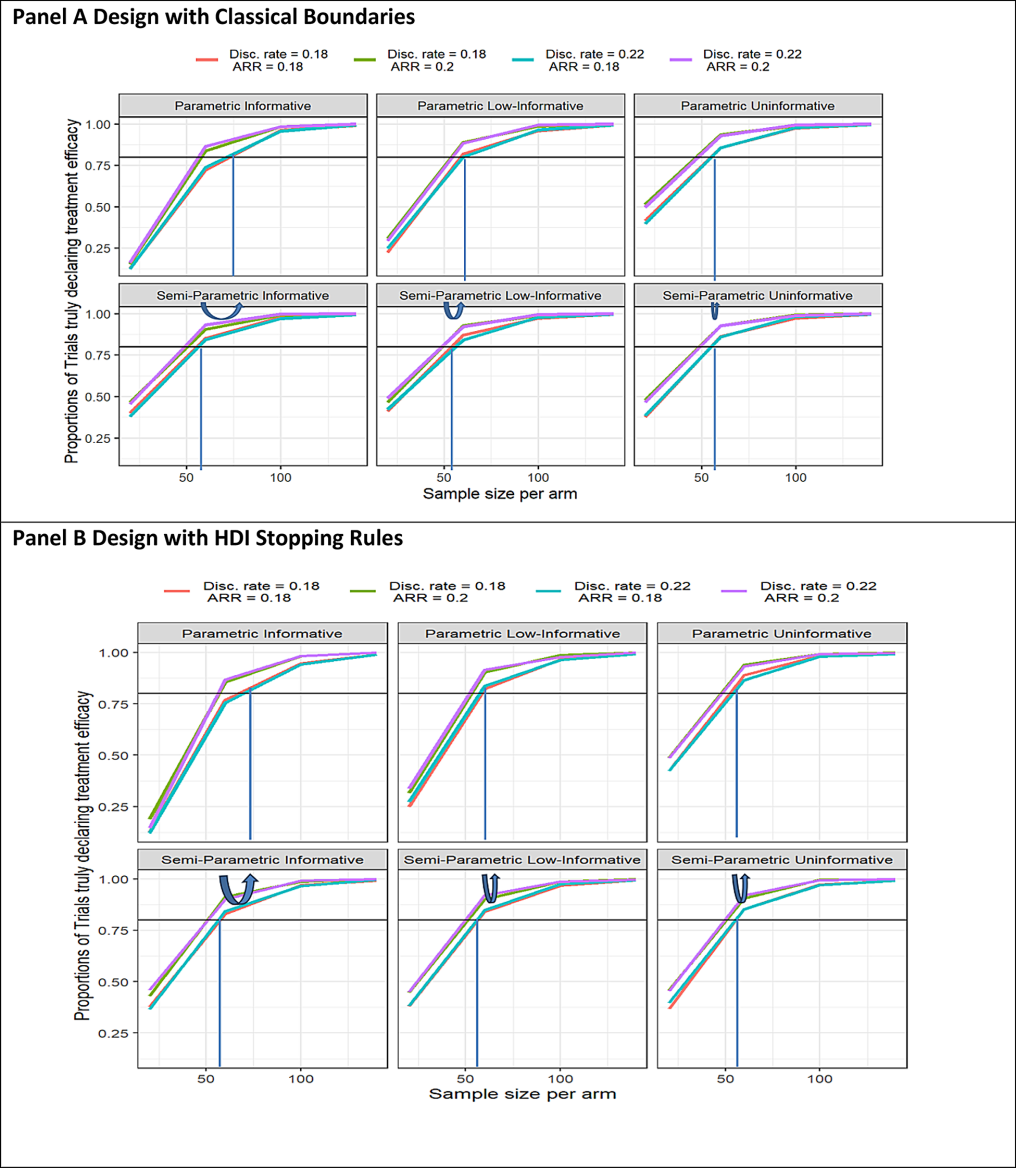
Proportions of simulated trials declaring the treatment effect, ad interim or at the end of the study, according to the sample size, simulation scenarios, and Prior Distributions

The Semiparametric prior design requires a smaller sample size to achieve a target power of 0.8 compared to Parametric methods when using informative priors. The higher sample size requirement for the informative prior results from prior-data conflict. When observed data deviate from prior assumptions, additional data are required to reconcile the posterior distribution and meet the power target. This effect is less pronounced for low-informative and uninformative priors(Fig. 2). Overall, power is lower for Parametric priors but becomes similar to Low-Informative and Uninformative priors (Appendix B, Table S2 1).

**Fig. 3:**
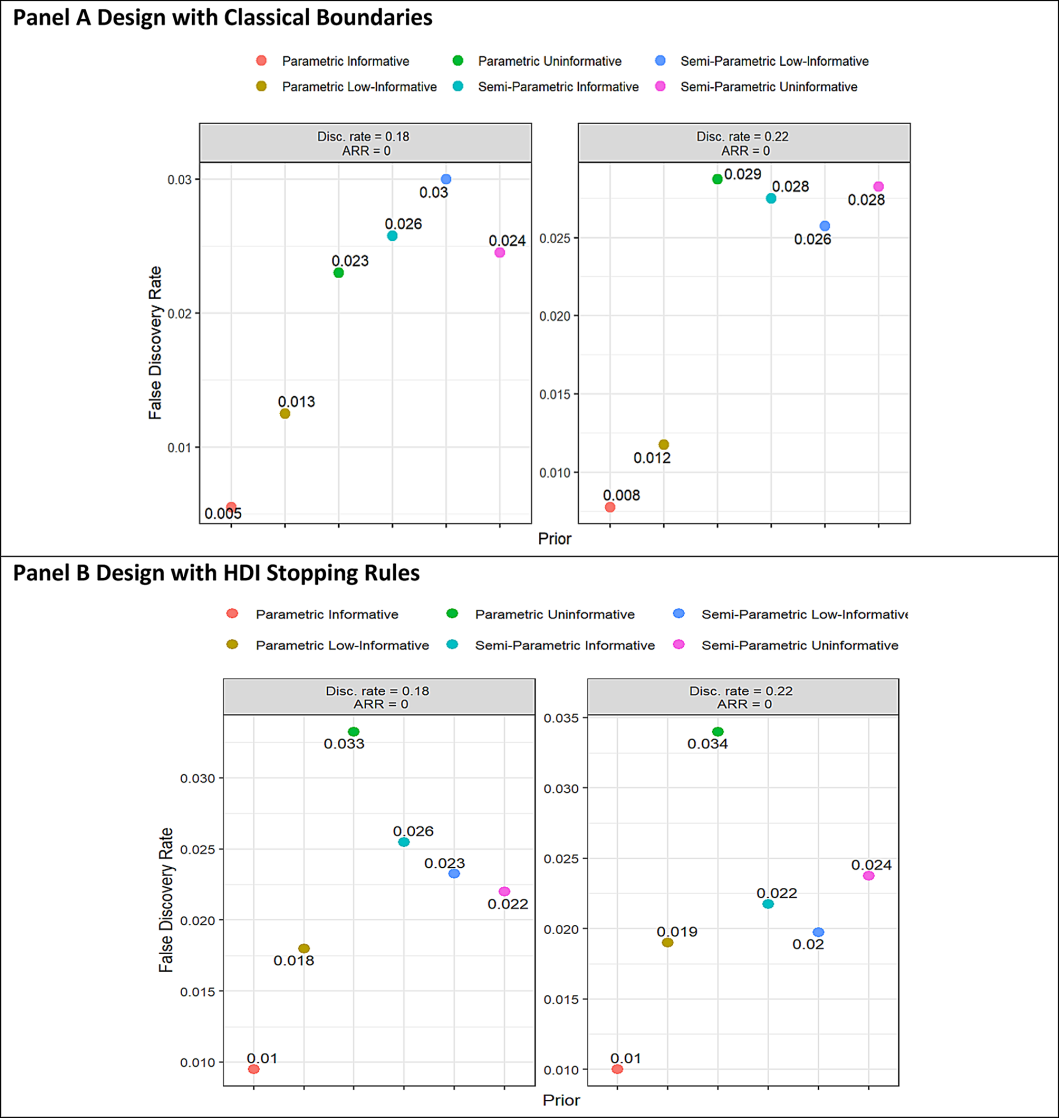
Average false discovery rate (FDR) over the sample size per simulation scenarios, and prior distributions

The false discovery rate (FDR) averages were within or below 0.035 for all the scenarios (Fig. 3). Design with Semiparametric priors have a slightly higher FDR than Parametric ones under classical stopping rules (Fig. 3, Panel A), but a lower FDR with HDI-based stopping rules compared to design using Parametric uninformative priors (Fig. 3, Panel B).

**Fig. 4:**
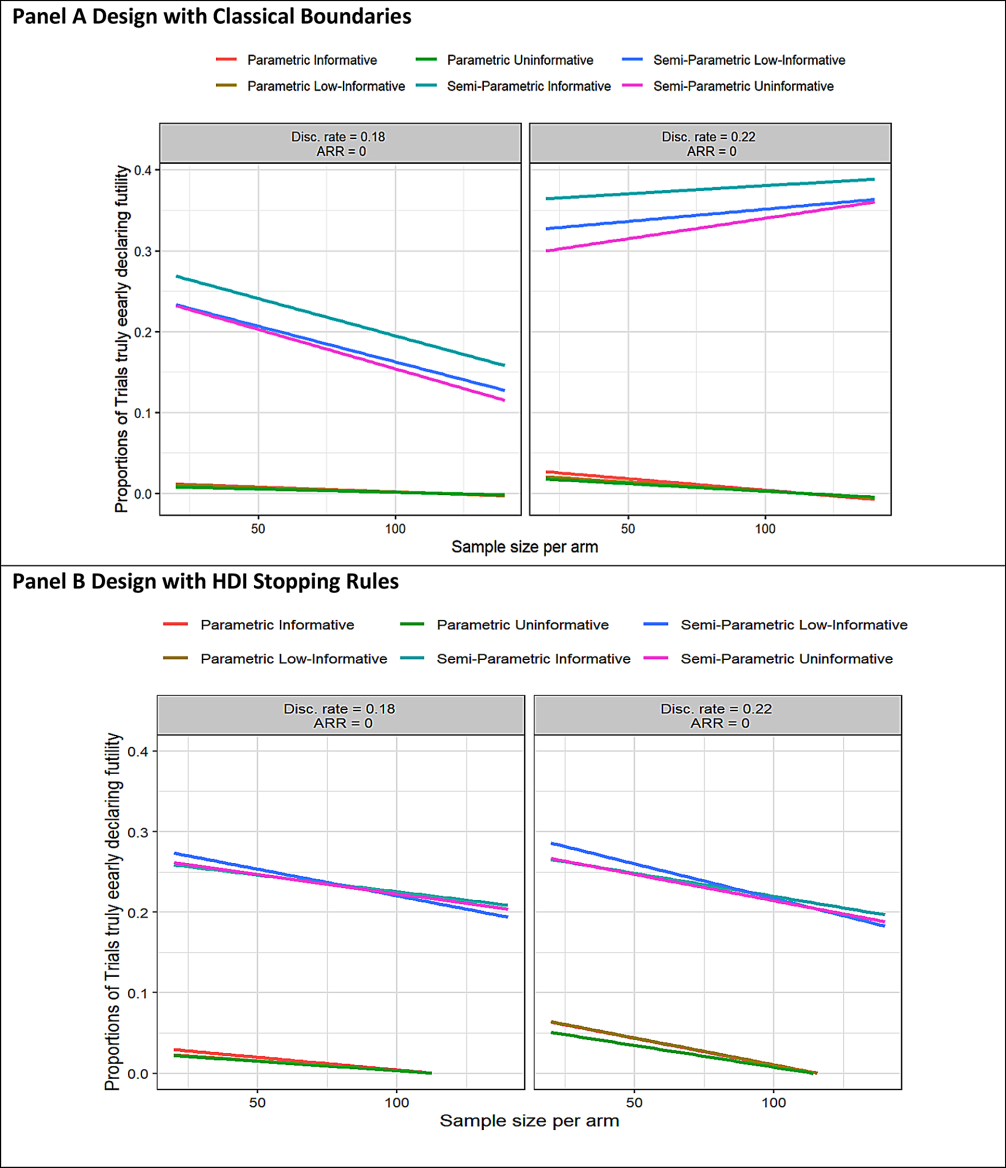
Proportions of simulated trials truly early declaring the futility ad interim according to the sample size, simulation scenarios, and prior distributions

In Semiparametric prior designs, the uninformative approach showed a greater tendency (over 10% of trials) to correctly reject early futility (Fig. 4). With classical stopping rules, this tendency increases with sample size if the discontinuation rate exceeds 0.2 and decreases otherwise (Fig. 4, Panel A). Using HDI-based stopping rules, the proportion decreases with sample size in all scenarios (Fig. 4, Panel B).

The proportion of trials falsely declaring futility in interim assessments was lower for Parametric prior elicitation methods and decreased with larger sample sizes (Appendix B, Figure S2 1). This difference narrows with uninformative approaches. Semiparametric prior elicitation with HDI maintains this rate below 5%, even for small samples, whereas it reaches 10% with classical stopping rules (Appendix B, Figure S2 1).

The frequency of trials correctly identifying efficacy at interim assessments was higher for Parametric priors and increased with sample size when using classical stopping rules (Appendix B, Figure S2 2, Panel A). With HDI-based stopping rules, the probability of correctly rejecting interim assessments increases if the safety event rate is below the acceptability threshold (Appendix B, Figure S2 2, Panel B).

## Discussion

Our study highlights the comparative efficacy of Parametric and Semiparametric prior elicitation methodologies in Bayesian adaptive clinical trials, with a focus on pediatric research. This demonstrates the advantages of a Bayesian sequential design with Semiparametric priors, particularly in terms of adaptability and responsiveness to diverse data landscapes typical of pediatric conditions. This flexibility is especially beneficial in scenarios with limited sample size or prior-data conflicts, which are common in pediatric research, particularly for rare diseases or conditions [34].

Prior-data conflict is a common challenge in pediatric trials, where historical data or expert opinions may not align with the emerging trial data [35]. Semiparametric priors stand out in their capacity to reconcile these discrepancies. Unlike traditional Parametric elicitation, which may struggle with incongruent prior information, Semiparametric priors can flexibly integrate and update beliefs in light of new evidence by increasing the ability to identify the treatment effect in an uncertain situation [16]. This is particularly important in pediatric research, where evolving understanding and limited previous studies can lead to initial assumptions that are at odds with real-world data [34]. The motivating example reported in this study highlights the importance of adaptability in pediatric clinical research. The proposed Semiparametric prior design integrates and updates beliefs using new evidence, making it beneficial in trials with incongruent prior information. In contexts like the RESCUE trial, this adaptability is useful for overcoming recruitment challenges and making informed decisions with limited data, prioritizing the ability to identify treatment effects in uncertain situations [30].

When the observed data conflicts with the prior assumptions, more data are required to reconcile the posterior distribution with the evidence from the trial using informative priors. This increases the sample size needed to achieve the target power. In contrast, low-informative and uninformative priors impose less constraint on the posterior, allowing the observed data to dominate the inference more rapidly, thereby requiring fewer patients to reach the desired power. In our findings, the Semiparametric prior design shows a need for a smaller sample size to achieve the target power compared to conventional Parametric elicitation when using informative priors, even in cases of prior data conflict, under both Classical and HDI-based stopping rules. This finding highlights the potential efficiency of Semiparametric elicitation in pediatric trials, where ethical considerations and limited patient populations make smaller sample sizes desirable even if uncertainty arises in the design phase [24, 36].

The average FDR remained within or below 0.035 across all sample sizes and scenarios, demonstrating the robustness of the Semiparametric solutions in pediatric trials. Although slightly higher under classical stopping rules, Semiparametric solutions achieve a lower FDR with HDI-based stopping rules compared with Parametric uninformative prior. This consistency in maintaining a low FDR underscores the reliability of our methodology in avoiding false positives, which is a crucial consideration in clinical trial designs [37]. The false discovery rate (FDR) observed in the simulations is influenced by multiple factors, including stopping rules, the degree of prior discounting, and the alignment of the observed data with the prior assumptions. The informative prior, defined by the expert opinions, reports a higher mean event rate compared to the control $(\:{\pi\:}_{T}-{\pi\:}_{C}>0)$ as reported in Appendix A. In contrast, the simulated data assumes equal event rates $(\:{\pi\:}_{T}-{\pi\:}_{C}=0)$ under the no effect assumption. The efficacy-stopping rule requires lower event rates for the treatment group compared to the control ($\:{\pi\:}_{T}-{\pi\:}_{C}<0$) to declare an effect. When the prior is highly influential (informative), it introduces a stronger contrast with the efficacy rule, making it more challenging to declare efficacy. Reducing the informativeness of the prior allows the observed data ($\:{\pi\:}_{T}-{\pi\:}_{C}=0$) to have greater influence, making it comparatively easier to declare efficacy under such conditions. These findings emphasize the important role of prior strength in Bayesian decision-making frameworks. Informative priors reduce variability and strongly anchor the posterior distribution to their initial assumptions, thereby significantly influencing the interpretation of the results. Reducing prior informativeness increases the influence of the observed data, thereby reducing bias but potentially increasing variability in posterior estimates. For this reason, priors should be chosen based on the available knowledge and alignment with expected data. In cases of high uncertainty, less informative priors may be preferable, to avoid anchoring posterior distributions to incorrect assumptions [24].

Semiparametric designs with uninformative approaches improve early rejection for futility compared to Parametric designs, especially with larger sample sizes under classical stopping rules if the discontinuation rate exceeds the safety threshold. Using HDI-based stopping rules, the early rejection decreased with the sample size in all scenarios. This finding is ethically advantageous in pediatric trials, as early identification of futility reduces young patients’ exposure to ineffective treatments and potential adverse events, upholding ethical standards and safeguarding their well-being [38]. Moreover, reducing unnecessary trial extensions and patient exposure can significantly lower trial costs [39].

In pediatric trials, where long-term treatment adherence and tolerability are important, dynamic adjustment to endpoints can significantly impact the assessment of treatment viability and safety. For example, in a pediatric oncology trial, the primary endpoint was typically the remission rate, while the discontinuation rate due to adverse effects, a key secondary endpoint, directly affected long-term adherence and quality of life [40]. In such trials, interim analysis with Semiparametric priors might reveal a promising increase in remission rates but also an unexpectedly high discontinuation rate due to severe side effects. For example, a significant number of young patients may have to discontinue treatment, exceeding the trial’s predetermined acceptable threshold. This critical information could lead to delayed responses in a conventional trial design, potentially continuing the trial without immediate modifications [41]. However, the Semiparametric elicitation allows the trial steering committee to quickly reassess the benefit-risk profile of the treatment. Given the high discontinuation rate, the trial could be adapted by modifying the dosage, changing administration methods, or even halting early, if necessary. This flexibility is relevant in pediatric oncology, where maintaining patients’ quality of life is as important as treating the disease [42].

Our findings suggest that Parametric priors more frequently identify efficacy in interim assessments, with this frequency increasing with sample size under classical stopping rules. With HDI-based stopping rules, correct rejection at interim assessments increases with the sample size only if the safety event rate is below the threshold. Thus, an informative Parametric prior is suitable when preliminary data are available and recruitment issues are unlikely. For example, a new pediatric trial evaluating asthma medication could benefit from extensive clinical or preclinical evidence by adopting a Parametric prior design [43].

The Semiparametric prior design shows advantages in sample size efficiency and managing FDR, especially with informative priors and HDI-based stopping rules, if uncertainty and prior data conflicts arise. Parametric elicitation design, while yielding higher interim power and correct efficacy identification rates, may require larger sample sizes. The choice between the approaches should consider the specific context, particularly in pediatric trials, where ethical considerations and limited data necessitate more conservative and informed decision-making.

### Limitations and future research developments

In this study, the simulation scenarios were specifically designed to reflect realistic clinical settings, based on the actual data from the RESCUE trial and informed through a real expert elicitation process. The choice of parameters considered for the simulations is performed to closely replicate the conditions that clinicians are likely to encounter in similar pediatric trials. This approach reinforces the clinical relevance of our findings.

However, we recognize the value of extending our analysis to include a wider array of scenarios in future studies. Such research could explore how our proposed method performs under different clinical conditions and parameter variations, potentially increasing its utility and applicability across a broader range of clinical settings. Future studies could also involve collaborations with clinicians to gather more extensive expert opinions, which may help in defining a wider range of plausible scenarios for further simulations. Additionally, incorporating real-world data from multiple clinical settings could help validate and refine our proposed statistical methods, ensuring they are robust across various pediatric populations and treatment conditions.

## Conclusion

This study presents a Bayesian adaptive approach for pediatric clinical trial design, emphasizing the effectiveness of Semiparametric prior elicitation. Our findings show that these methods are particularly useful for handling the complexities of pediatric trials, where prior data may be sparse or conflicting. The adaptability of Semiparametric prior in updating new evidence is useful in facing recruitment challenges and requiring informed decisions with limited data.

## Electronic supplementary material

Below is the link to the electronic supplementary material.

Supplementary Material 1

Supplementary Material 2

## Data Availability

No datasets were generated or analysed during the current study.
