# Supplementary material for: Navigating challenges in pediatric trial conduct: integrating bayesian sequential design with semiparametric elicitation for handling primary and secondary endpoints
Source: BMC Med Res Methodol. 2025 Mar 31;25:82. doi: 10.1186/s12874-025-02484-7 (PMC11956446; doi:10.1186/s12874-025-02484-7)
Supplement: Supplementary file 1 — Supplementary Material 1 [file 12874_2025_2484_MOESM1_ESM.docx]

**Appendix B**

**Figure S2 1** Proportion of simulated trials falsely declaring the futility at interim assessment according to the sample size, simulation scenarios, and Prior Distributions.

| **Panel A Design with Classical Boundaries**  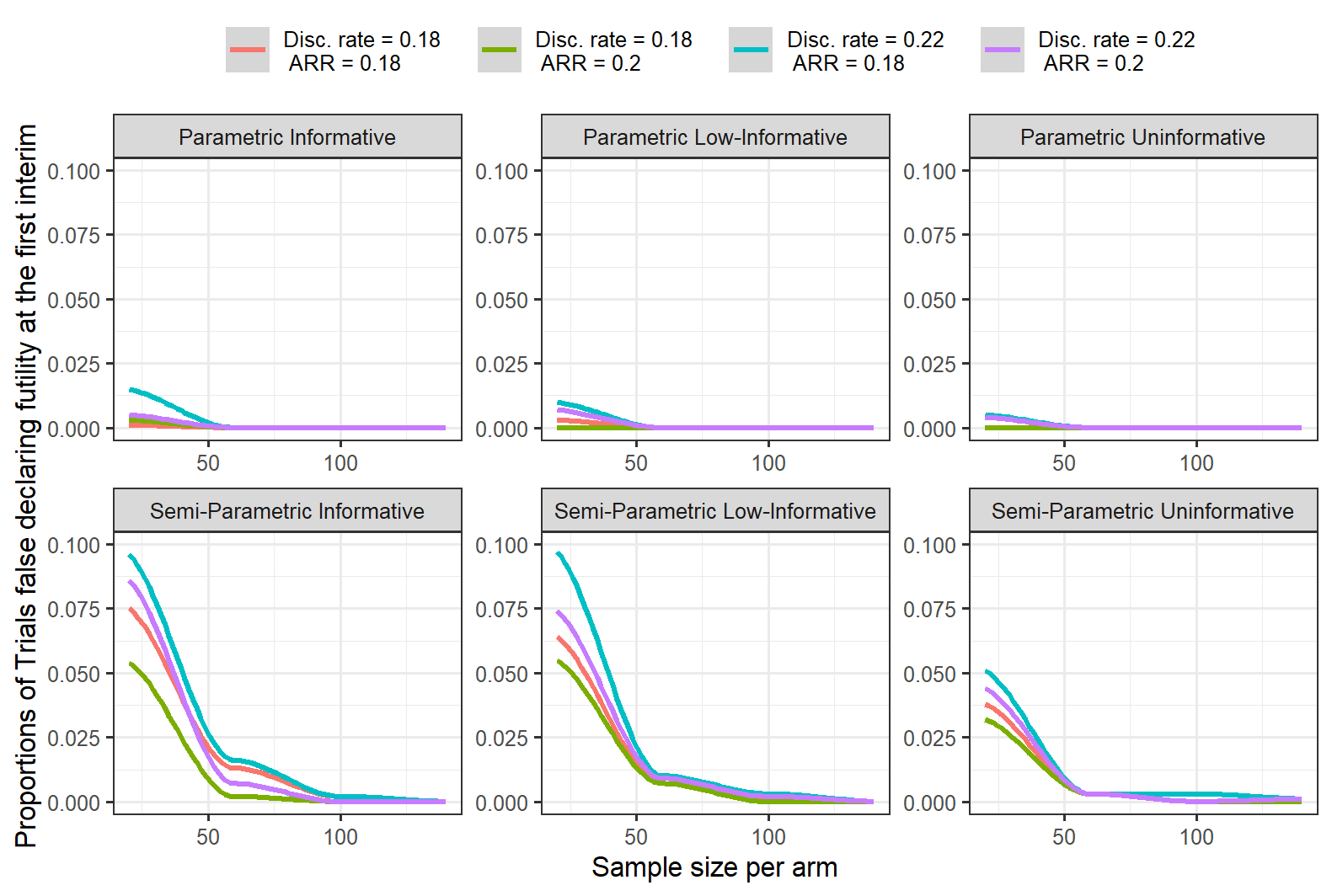 |
| --- |
| **Panel B Design with HDI Stopping Rules**  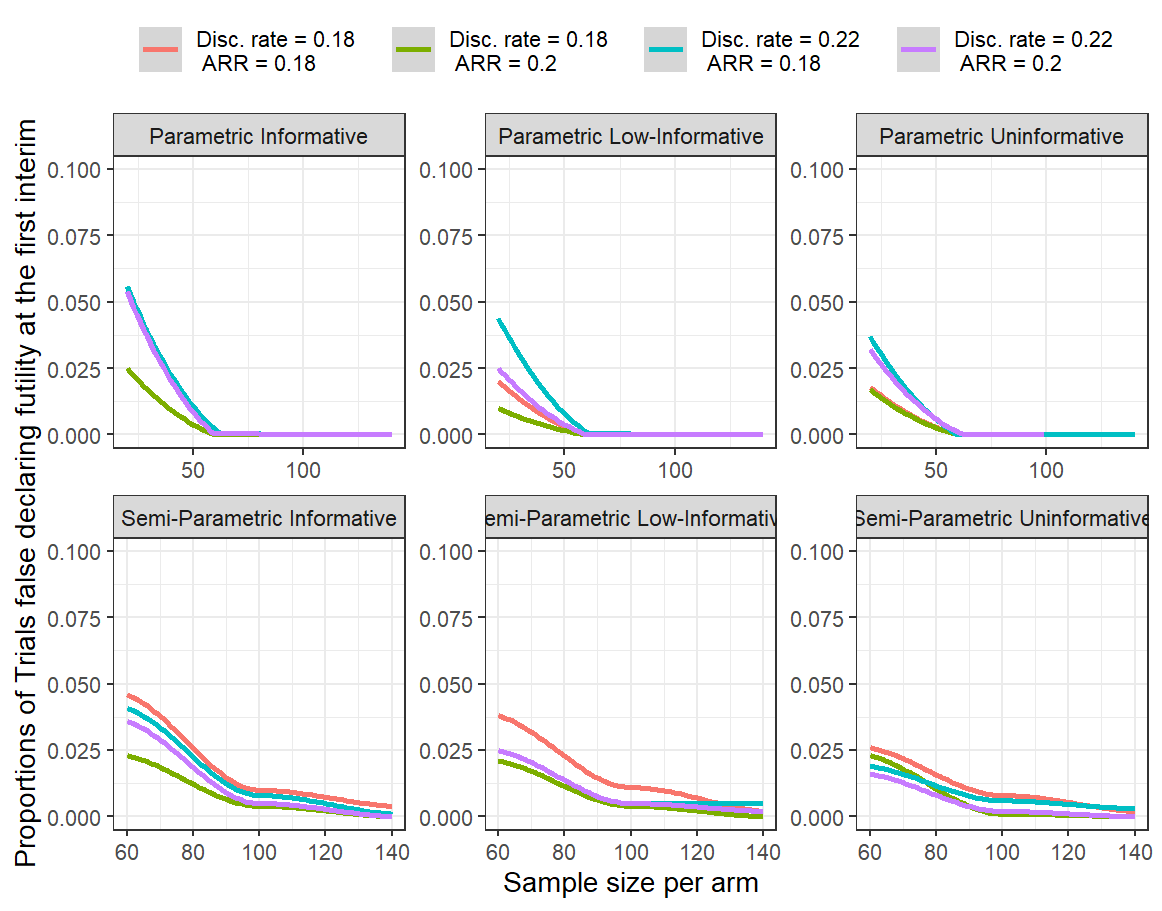 |

**Figure S2 2** Proportions of simulated trials truly declaring the efficacy at interim assessment according to the sample size, simulation scenarios, and Prior Distributions.

| **Panel A Design with Classical Boundaries**  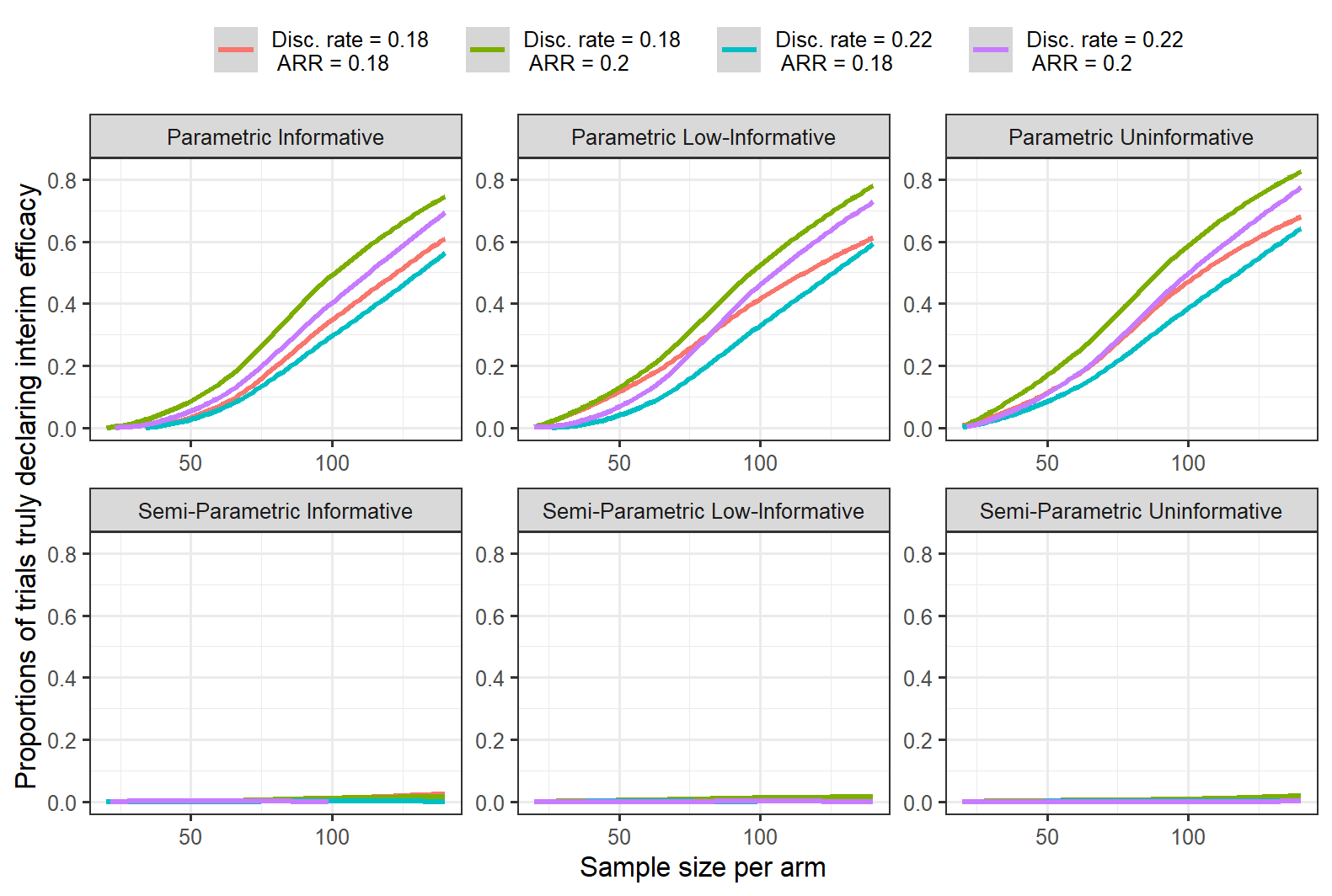 |
| --- |
| **Panel B Design with HDI Stopping Rules**  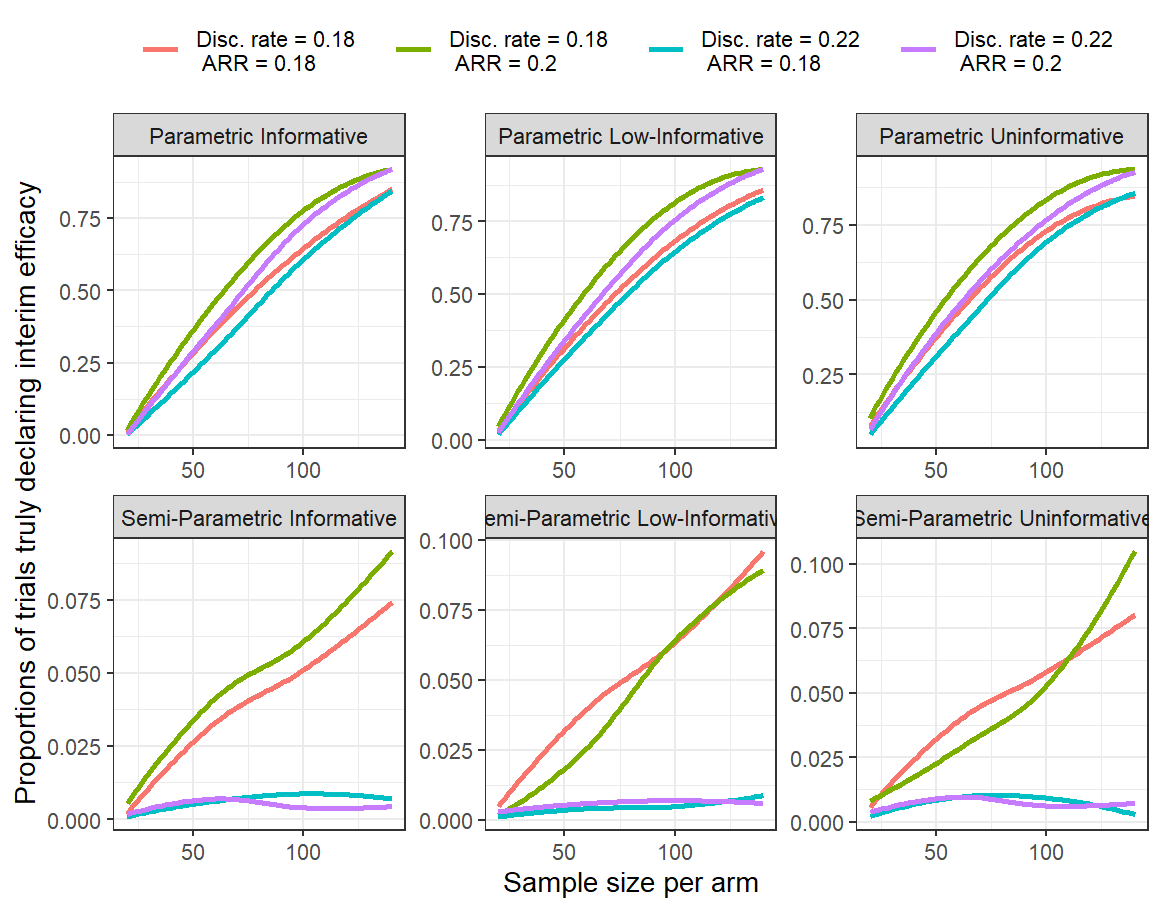 |

**Table S2 1** Overall Power, rate of trials falsely declaring the futility and truly declaring the efficacy at interim assessment averaged over the sample size. Results are reported over simulation scenarios and Prior Distributions.

|  |  | **Design with Classical Boundaries** | | | | | | **Design with HDI Stopping Rules** | | | | | | |
| --- | --- | --- | --- | --- | --- | --- | --- | --- | --- | --- | --- | --- | --- | --- |
|  |  | **Priors** | | | | | | **Priors** | | | | | | |
|  |  | **Parametric** | | | **Semiparametric** | | | **Parametric** | | | | **Semiparametric** | | |
|  | **Simulation Scenarios** | **Power** | **False interim futility Rate** | **True interim efficacy Rate** | **Power** | **False interim futility Rate** | **True interim efficacy Rate** | **Power** | **False interim futility Rate** | **True interim efficacy Rate** | **Power** | | **False interim futility Rate** | **True interim efficacy Rate** |
| **Informative** | Disc. rate = 0.18   ARR = 0.18 | 0.7005 | 0.0003 | 0.2573 | 0.8063 | 0.0225 | 0.0090 | 0.7083 | 0.0063 | 0.4678 | 0.7920 | | 0.0625 | 0.0400 |
|  | Disc. rate = 0.18   ARR = 0.2 | 0.7433 | 0.0008 | 0.3448 | 0.8405 | 0.0140 | 0.0073 | 0.7570 | 0.0063 | 0.5438 | 0.8328 | | 0.0453 | 0.0498 |
|  | Disc. rate = 0.22   ARR = 0.18 | 0.7035 | 0.0038 | 0.2295 | 0.7968 | 0.0285 | 0.0005 | 0.7025 | 0.0145 | 0.4370 | 0.7930 | | 0.0555 | 0.0058 |
|  | Disc. rate = 0.22   ARR = 0.2 | 0.7525 | 0.0013 | 0.2988 | 0.8460 | 0.0233 | 0.0005 | 0.7493 | 0.0135 | 0.5075 | 0.8378 | | 0.0460 | 0.0043 |
| **Low-Informative** | Disc. rate = 0.18   ARR = 0.18 | 0.7488 | 0.0008 | 0.2988 | 0.8123 | 0.0180 | 0.0068 | 0.7580 | 0.0050 | 0.4925 | 0.7955 | | 0.0575 | 0.0510 |
|  | Disc. rate = 0.18   ARR = 0.2 | 0.7955 | 0.0000 | 0.3743 | 0.8455 | 0.0155 | 0.0080 | 0.8020 | 0.0025 | 0.5765 | 0.8340 | | 0.0425 | 0.0453 |
|  | Disc. rate = 0.22   ARR = 0.18 | 0.7515 | 0.0025 | 0.2498 | 0.8095 | 0.0275 | 0.0003 | 0.7673 | 0.0113 | 0.4635 | 0.8005 | | 0.0533 | 0.0048 |
|  | Disc. rate = 0.22   ARR = 0.2 | 0.7923 | 0.0018 | 0.3273 | 0.8508 | 0.0213 | 0.0003 | 0.8080 | 0.0063 | 0.5365 | 0.8390 | | 0.0445 | 0.0055 |
| **Uninformative** | Disc. rate = 0.18   ARR = 0.18 | 0.8113 | 0.0010 | 0.3315 | 0.8018 | 0.0103 | 0.0050 | 0.8240 | 0.0045 | 0.5290 | 0.7965 | | 0.0453 | 0.0458 |
|  | Disc. rate = 0.18   ARR = 0.2 | 0.8590 | 0.0001 | 0.4140 | 0.8490 | 0.0088 | 0.0078 | 0.8550 | 0.0043 | 0.6083 | 0.8403 | | 0.0383 | 0.0485 |
|  | Disc. rate = 0.22   ARR = 0.18 | 0.8065 | 0.0013 | 0.2900 | 0.8055 | 0.0145 | 0.0013 | 0.8158 | 0.0093 | 0.4980 | 0.8028 | | 0.0420 | 0.0060 |
|  | Disc. rate = 0.22   ARR = 0.2 | 0.8553 | 0.0010 | 0.3600 | 0.8455 | 0.0120 | 0.0003 | 0.8523 | 0.0083 | 0.5603 | 0.8423 | | 0.0348 | 0.0068 |
