# Supplementary material for: Navigating challenges in pediatric trial conduct: integrating bayesian sequential design with semiparametric elicitation for handling primary and secondary endpoints
Source: BMC Med Res Methodol. 2025 Mar 31;25:82. doi: 10.1186/s12874-025-02484-7 (PMC11956446; doi:10.1186/s12874-025-02484-7)
Supplement: Supplementary file 2 — Supplementary Material 2 [file 12874_2025_2484_MOESM2_ESM.docx]

# **Appendix A**

# Prior distributions

## Expert Elicitation

During the planning phase of the RESCUE trial, there was no concrete data to establish a prior distribution. Consequently, an elicitation study was carried out to gather expert opinions and form a prior distribution.

To achieve this, experts were given a questionnaire aimed at deriving a prior distribution for the likelihood of adverse events in children suffering from a particular illness. Moreover, all the experts participating in this study routinely employ the treatment in question in their clinical work.

As required in the commonly used SHELF elicitation procedure[1], a joint expert evaluation session was difficult to perform in this research setting. Furthermore, the involvement of several experts simultaneously in the same elicitation session was not easy to implement for practical reasons (related to the different organization of work among experts, the location of work, the different shifts, etc.). Therefore, a single expert guessing approach was used; the information among the experts was combined by taking the average of guesses and calculating the variance. However, this approach has previously been acknowledged and utilized in other contexts within existing literature [2, 3].

The questions posed to the experts for eliciting the renal Scar probabilities respectively in the control and in the treatment, arms are:

1. *“Based on your experience, what is the probability that a patient aged 0 to 2, with a value of procalcitonin >1 µg/L, treated with the recommended antibiotic regimen, has evidenced the presence of a renal scar event 6 months after the acute episode?”*
2. “Based on your experience, what is the probability that a patient aged 0 to 2, with a value of procalcitonin >1 µg/L, treated with the recommended antibiotic regimen+dexametasone, has evidenced the presence of a renal scar event 6 months after the acute episode?”

The experts responding to the questions are 8 for the control arms and 5 for the treatment arm (Table S1 1).

**Table S1 1** Expert opinion for the scar rate probability in the treatment and the control arm. The mean $\mu$ and standard deviation $\sigma$ of the opinions have been reported together with the corresponding $\alpha_{0}$ shape and rate $\beta_{0}$ parameters for a Beta random variable

| **Expert** | **Opinion Control** | **Opinion Treatment** |
| --- | --- | --- |
| **1** | 0.3 | 0.5 |
| **2** | 0.25 | 0.25 |
| **3** | 0.15 | 0.3 |
| **4** | 0.4 | 0.5 |
| **5** | 0.3 |  |
| **6** | 0.2 |  |
| **7** | 0.2 | 0.3 |
| **8** | 0.3 | 0.25 |
|  |  |  |
| $\mu$ | **0.26** | **0.35** |
| $\sigma$ | 0.08 | 0.12 |
| $\alpha_{0}$ | 8 | 5 |
| $\beta_{0}$ | 22 | 10 |

Informative, Low-Informative, and Uninformative prior scenarios are considered for computation using a parametric Beta prior and a semiparametric solution.

For the Beta prior, the parameters $\alpha_{0}$ and $\beta_{0}$are determined from the expert's estimated mean and variance using the formula:

$$\alpha_{0}=\left( \frac{(1-\mu)}{\sigma^{2}}-\frac{1}{\mu} \right)\mu^{2}\beta_{0}=\alpha_{0}\left( \frac{1}{\mu}-1 \right)$$

These formulas convert the mean and variance into a shape that reflects both the central tendency and the dispersion of the expert's beliefs about the parameter of interest (Table S1 1).

## Parametric approach

In the context of the Beta parametric framework, the degree to which the prior affects the final estimation was determined using a power prior methodology [4].

Various degrees of discounting can be applied to the expert opinion to conduct a sensitivity analysis regarding the choices of the prior. The expert's input can be factored into the final calculation using a $Beta(\alpha,\beta)$ prior, where:

$$\begin{aligned} &\alpha=\alpha_{0}d_{0}+1 \\ &\beta=\beta_{0}d_{0}+1 \end{aligned}$$

The parameters $\alpha_{0}$ $\mathrm{and} \beta_{0}$ are derived from the mean $\mu$ and variance $\sigma^{2}$of the expert opinions (Table S1 1), utilizing a reverse calculation formula, where:

$$\begin{aligned} \alpha_{0}=\left[ \left( \frac{1-\mu}{\sigma^{2}}-\frac{1}{\mu} \right)\mu^{2} \right]-1 \\ \beta_{0}=\left[ \alpha\left( \frac{1}{\mu}-1 \right) \right]-1 \end{aligned}$$

The parameter $d_{0}$determines the proportion of expert opinion contributing to the final design via prior distribution. Conversely, the discounting factor is calculated as$({1-d}_{0})\times100$, indicating the extent to which the expert opinion is discounted or penalized.

- When$d_{0}$ = 0 equals 0, which implies that the information provided by the expert opinion is entirely disregarded, equating to a 100% discount on the prior information. In such a case, the prior defaults to an uninformative $Beta(1,1)$ distribution.
- If $d_{0}$=1, it means that all of the experts' information is fully incorporated into the inference process, resulting in no discounting (0%) of the expert opinion.

## Semiparametric method

The prior distribution for rates can be obtained through a semiparametric method by eliciting opinions from experts [5]. Within this structure, the prior distribution is derived by optimizing a weighted combination of two elements:

1. A component evaluating how well a prior distribution aligns with the quantiles determined by experts.
2. The measure of how far the prior is from a uniform uninformative distribution.

A uniform distribution is used as the benchmark for an uninformative prior, in line with recommendations found in existing literature [5], to achieve a prior distribution that effectively balances being completely uninformative and being an informative function tailored to fit expert quantiles.

The shape of the probability density function, as informed by expert quantiles, is approximated through a linear combination of B-splines. These B-splines have inner knots that align with specified boundaries. In this theoretical approach, *F* represents a spline of *j* degree, incorporating a sequence of *S* inner knots $\lambda=\left( \lambda_{-m},\ldots,\lambda_{S+j+1} \right)^{T}$. Following guidelines in the literature, certain constraints are applied to ensure that this linear combination forms a valid density function [5].

Given p elicited quantiles $y_{\alpha_{1}},\ldots,y_{\alpha_{p}}$, modeled by a linear combination of B-splines, the expert density function can be ascertained by optimizing the following objective function where$f$ represents the target prior density, and $F$ is associated cumulative distribution function.

$$\min_{F_{-j},\ldots,F_{S}}\left\{ \sum_{i=1}^{p} \left( \alpha_{i}-F\left( y_{\alpha_{i}} \right) \right)^{2}+k\int_{y_{0}}^{y_{1}} f(y)^{2}dy \right\}$$

$$F_{i}\leq F_{i+1}\text{ for }i=-j,\ldots,S-1$$

$$\text{and }F_{-j}=0,F_{S}=1$$

The balancing factor $k$ can be determined by setting a predefined expected $\Delta$ error, which represents the discrepancy between the expert distribution and the established p quantiles.

$$\Delta=\sqrt{\frac{1}{p}\sum_{i=1}^{p} \left( \alpha_{i}-F\left( y_{\alpha_{i}} \right) \right)^{2}}$$

This method aims to capture a true-to-life depiction of the expert's uncertainty. In cases where a specific statement of uncertainty is not accessible, it's possible to employ standard default values for $\Delta ADDIN ZOTERO\_ITEM CSL\_CITATION \{"citationID":"j4SBjwvG","properties":\{"formattedCitation":"[5]","plainCitation":"[5]","noteIndex":0\},"citationItems":[\{"id":23735,"uris":["http://zotero.org/groups/2201980/items/KKL58JB4"],"itemData":\{"id":23735,"type":"article-journal","container-title":"The American Statistician","DOI":"10.1198/tast.2009.08191","issue":"4","page":"373-377","title":"A note on B-splines for semiparametric elicitation","volume":"63","author":[\{"family":"Bornkamp","given":"Björn"\},\{"family":"Ickstadt","given":"Katja"\}],"issued":\{"date-parts":[["2009"]]\}\}\}],"schema":"https://github.com/citation-style-language/schema/raw/master/csl-citation.json"\} [5]$. The authors recommend determining this default $\Delta$ through a data-driven strategy. In this context, the declared quantiles $y_{\alpha_{i}}$ are normalized ($y_{{\alpha_{i}}^{*}}$) in relation to the boundary bounds $\left[ j,k \right]$of the parametric space being examined.

$$y_{{\alpha_{i}}^{*}} =(y_{\alpha_{i}}-j)/(k-j)$$

The data-derived $\Delta^{*}$ is computed as a quadratic loss function, which involves comparing the standardized stated quantiles $y_{{\alpha_{i}}^{*}}$ with the numerical vector that specifies the levels of the quantiles $y_{i}$:

$$\Delta^{*}=\left( \sqrt{\frac{1}{p}\sum_{i=1}^{p} \left( y_{{\alpha_{i}}^{*}}-y_{i} \right)^{2}} \right)/2$$

For the expert elicitation process, the quartiles 0.25, 0.5, and 0.75 were selected from the expert opinions. This method is among the more prevalent techniques for eliciting fractiles [6].

## Informative priors

The prior distribution on the secondary endpoint is defined as a $Beta(1,1)$ variable.

The expert opinions are instead used on the treatment effect to obtain informative prior probability distribution in a parametric or semiparametric setting, considering:

1. A prior distribution$Beta(\alpha_{i},\beta_{i})$ with shape and scale obtained from the mean $\mu$ and variance $\sigma^{2}$ of the expert opinions
2. A B-spline semiparametric prior defined considering the inner knots located on the expert quartiles with j=4 degree of approximation for the B-Spline [5]. The author stated that similar results could be obtained with a smoother fit by increasing the degrees of approximation. The prior informativeness parameter, $k=0.13,$ is instead derived from $\Delta=0.146 as$ indicated in the literature [5].

## Low-Informative priors

Low-informative priors have been defined in the computation considering:

1. $Beta(\alpha_{i},\beta_{i})$ with a $d_{0}$=0.5 (50% discounting).
2. A B-splines semiparametric prior with *j=4* degrees and $k$=1.

## Uninformative priors

The uninformative priors have been compared with other scenarios, respectively in parametric and semiparametric settings deriving the following priors:

1. A prior distribution Beta (1,1) with $d_{0}$=0 (100% discounting);
2. A B-splines semiparametric prior defined considering the inner knots located on the quartiles defined by an expert with *j=4* degrees and $k$=45.

The prior distributions are reported in the Figure S1 2.

As highlighted in the literature[7], spline modeling offers substantial flexibility but requires careful selection of tuning parameters such as the choice of basis functions and the degree of the polynomials. The impact of these parameters is generally minimal; spline fits are robust to changes in the polynomial degree. Cubic polynomials (*j = 3*) are commonly used because they yield visually smooth curves. Although higher degrees can be employed for calculating derivatives of the curves, the visual distinction of fits with a degree higher than three is often negligible. Conversely, using a polynomial degree of one or two results in curves that, while statistically similar, tend to look more jagged. Following the guidance of Bornkamp and Ickstadt, we opted for a polynomial degree of four (*j = 4*), within the recommended range of two to five, and positioned the inner knots at the quantiles to enhance the flexibility at these specific points. In Figure S1 3 the shape of the prior remains consistent when comparing degrees three, four, and five.

**Figure S1 2** Prior distributions for the expert opinion for the scar rate probability in the treatment and the control arm. The Parametric and semiparametric priors have been considered with several discounting factors. The first two moments (mean and variance) of scar rate distributions for informative, low-informative, and uninformative priors in both parametric and semiparametric cases are displayed.


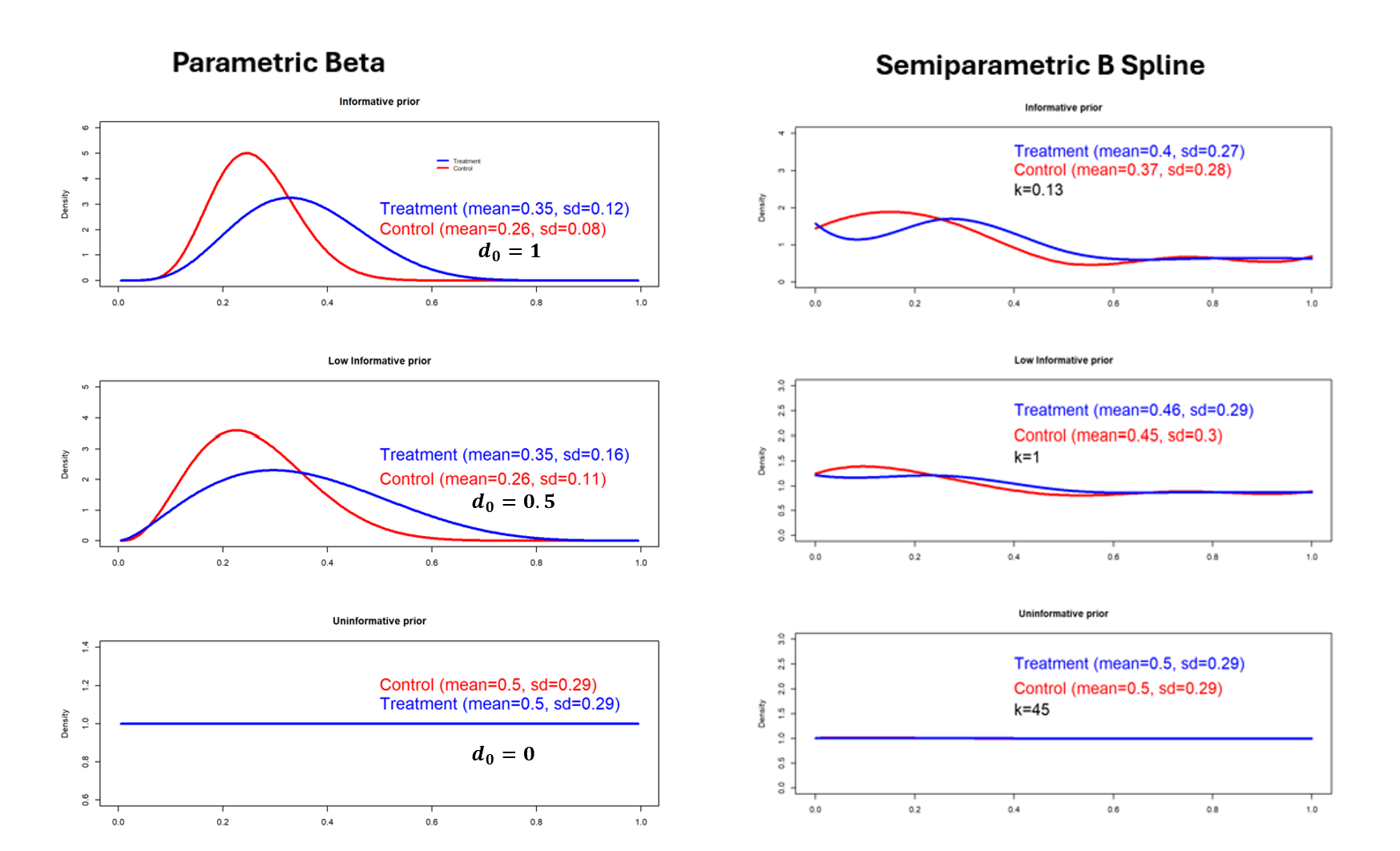


**Figure S1 3** Prior distributions for the expert opinion for the scar rate probability in the treatment and the control arm. The semiparametric priors have been considered with several discounting factors and degree of splines (*j=3, j=4, j=5*). The first two moments (mean and variance) of scar rate distributions for informative, low-informative, and uninformative priors are displayed.


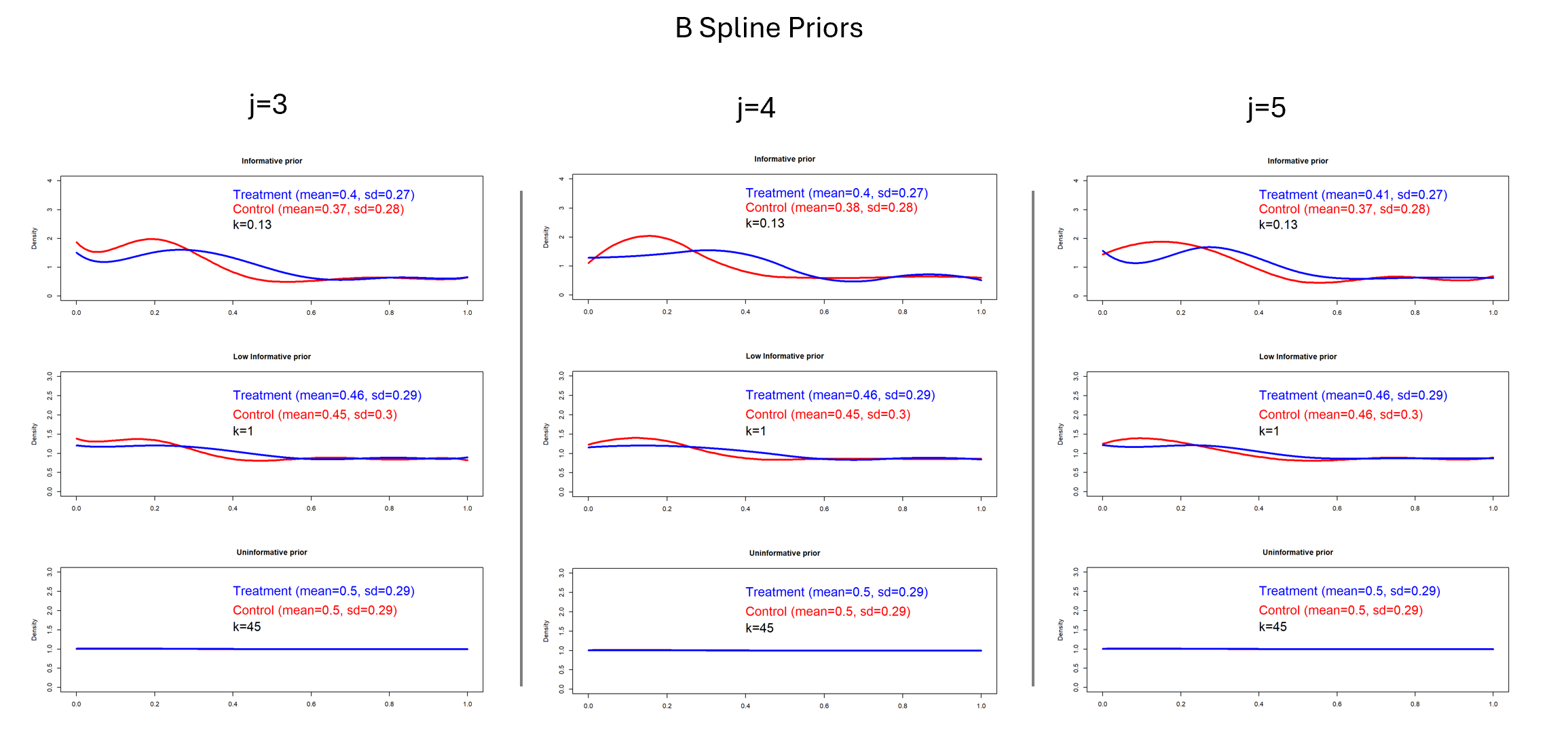


# Simulation Plan details

Several simulation scenarios were provided by mimicking the motivating example data-generation mechanism. The sample size has been assumed in the range $n=40,\ldots, 300$ per arm. The event rate in the control arm was assumed $\Pi_{C}=0.4$ as provided in the study protocol. The effect size was assumed as ${\Pi_{C\text{ }}-\Pi}_{\text{T }}=0, 0.18, 0.2$, similar to as observed in the original trial results[8]. The secondary outcome which is the probability of discontinuation [8] is assumed $\Pi_{\text{disc }}=0.18,\ldots0.22$ by assuming scenarios of discontinuation rates lower and higher than a tolerated level $m=0.2$, slightly higher than 0.18 as observed in the motivating example results[8] (Figure S1 4).

The data were generated in a situation of uncertainty in the study design phase, where a prior-data conflict scenario could arise, and the expert opinion about the treatment effect conflicts with the data simulation scenario. The experts are confident in observing a high scar rate in the treatment (0.35+-0.12) arm versus control (0.26+-0.08), as reported in the Table S1 1; in the data, instead, the control scar rate is assumed higher than the treatment, or a null effect is reported.

Each simulation scenario was generated 10,000 times and analyzed using six prior distributions from expert opinions (Appendix A, Table S1 1). The priors were as follows: 1) Parametric Beta Informative, 2) Parametric Beta Low-Informative, 3) Parametric Beta Uninformative, 4) Semiparametric B-Spline Informative, 5) Semiparametric B-Spline Low-Informative, and 6) Semiparametric B-Spline Uninformative (Figure S1 3).

The posterior distributions for each analysis were achieved via 50,000 MCMC (Markov Chain Monte Carlo) resampling (three chains). The control scar rate $\pi_{C}^{*}$ was resampled by the posterior $\pi_{C}|X_{C}$; the same for $\pi_{T}^{*}$ from $\pi_{C}|X_{C}$, to achieve the ARR outcome posterior ${ARR=\pi}_{T}^{*}- \pi_{C}^{*}$. The posterior probability of discontinuation is resampled from $\pi_{disc}^{*}$ from $\pi_{disc}|X_{disc}$.

**Figure S1 4** Simulation Plan


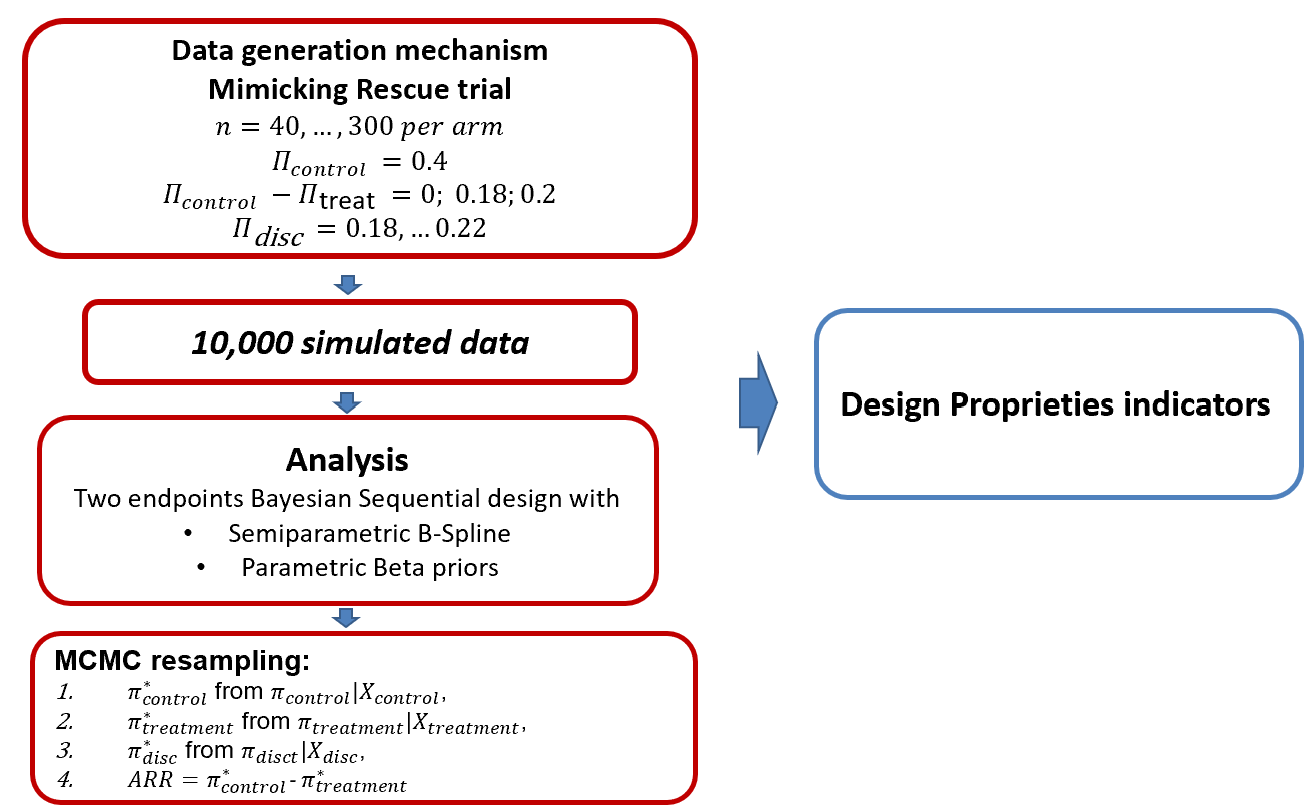


## References

1. Gosling JP. SHELF: The Sheffield Elicitation Framework. In: Dias LC, Morton A, Quigley J, editors. Elicitation. Cham: Springer International Publishing; 2018. p. 61–93.

2. Berchialla P, Zohar S, Baldi I. Bayesian sample size determination for phase IIA clinical trials using historical data and semi‐parametric prior’s elicitation. Pharm Stat. 2018.

3. Zohar S, Baldi I, Forni G, Merletti F, Masucci G, Gregori D. Planning a Bayesian early-phase phase I/II study for human vaccines in HER2 carcinomas. Pharm Stat. 2011;10:218–26.

4. Ibrahim JG, Chen M-H. Power prior distributions for regression models. Stat Sci. 2000;15:46–60.

5. Bornkamp B, Ickstadt K. A note on B-splines for semiparametric elicitation. Am Stat. 2009;63:373–7.

6. Garthwaite PH, Kadane JB, O’Hagan A. Statistical methods for eliciting probability distributions. J Am Stat Assoc. 2005;100:680–701.

7. Perperoglou A, Sauerbrei W, Abrahamowicz M, Schmid M. A review of spline function procedures in R. BMC Med Res Methodol. 2019;19:46.

8. Da Dalt L, Bressan S, Scozzola F, Vidal E, Gennari M, La Scola C, et al. Oral steroids for reducing kidney scarring in young children with febrile urinary tract infections: the contribution of Bayesian analysis to a randomized trial not reaching its intended sample size. Pediatr Nephrol. 2021. https://doi.org/10.1007/s00467-021-05117-5.
